# Functional-thermoregulatory model for the differential diagnosis of psoriatic arthritis

**DOI:** 10.1186/1475-925X-13-162

**Published:** 2014-12-11

**Authors:** Enas Ismail, Alessandra Capo, Paolo Amerio, Arcangelo Merla

**Affiliations:** Department of Neuroscience, Imaging and Clinical Sciences, University “G. d’Annunzio”, Via dei Vestini, 31, 66013 Chieti, Italy; ITAB -Institute of Advanced Biomedical Technologies, Via dei Vestini, 32, 66013 Chieti, Italy; Department of medicine and Aging Science, University “G. d’Annunzio”, Chieti-Pescara, Italy

**Keywords:** Control system, Cutaneous temperature, Modeling, Psoriatic arthritis, Thermal infrared imaging, Isometric exercise, Multinomial logistic regression

## Abstract

**Introduction:**

Psoriasis arthritis (PsA) is a chronic inflammatory arthritis of joints of uncertain pathogenesis. PsA may lead to severe disabilities even in the absence of any clinical symptom. Therefore, PsA diagnosis in its early stages is critical.

**Material and methods:**

This study uses Control System theory to model finger skin thermoregulatory processes overlying the hand joint in response to an isometric exercise. The proposed model is based on a homeostatic negative feedback loop characterized by four distinct parameters that describe how the control mechanisms are activated and maintained. Thermal infrared imaging was used to record a total of 280 temperature curves of 14 finger joints for each of 11 PsA patients and 9 healthy controls.

**Result and conclusion:**

PsA patients presented delayed and prolonged re-warming processes characterized by the undershoot onset after the end of the isometric exercise followed by a faster temperature increase. Region classification on the basis of the model parameters demonstrated that the interphalageal joint region of thumb better discriminates between patients and controls, providing 100% true-positive discrimination for PsA affected regions and 88.89% of correct classification of healthy regions. Even proved over a limited number of subjects, the proposed method may provide useful hints for early differential diagnosis in the IR assessment of PsA disease.

## Introduction

Psoriasis (PsO) is a chronic, complex, immuno-inflammatory disease involving the skin and the musculoskeletal structures [1]. Psoriasis arthritis (PsA) is a chronic inflammatory arthritis of uncertain pathogenesis that affects around 25% of worldwide psoriatic patients [1]. PsA commonly affects the tips of fingers and toes [2]. Psoriasis skin lesions typically precede the onset of joint symptoms, damage peripheral and axial joints by 10 months of symptom onset in around 27% of patients and 2 years of symptom onset in 47% of patients [1]. After that period, patients experience severe disabilities such as difficulty with grasping their hand [1, 2]. The diagnosis of PsA is not always immediate since there are not specific circulating markers and its symptoms are frequently unstable. Ultrasonography (US) and magnetic resonance (MRI) are considered the gold standard imaging methods for documenting clinical and sub-clinical PsA [1]. However, their use in clinical routine for early diagnosis of PsA may be limited by their cost (especially MRI) or dependency on the operator’ skill (especially US) [2, 3]. Local thermoregulatory malfunctions were found to be manifested by the presence of PsA disorder [4]. In fact, psoriatic skin vascular features may induce large thermal changes in skin temperature in psoriatic plaques [3–5], however little is known about the effect of the joint inflammatory process of PsA on normal skin overlying affected joints in PsA patients. Infrared (IR) imaging is a non-invasive diagnostic technique that is able to provide two-dimensional maps of the cutaneous temperature distribution of a given body by measuring emitted infrared energy [6, 7]. Moreover, since the cutaneous temperature depends on the local blood perfusion and thermal tissue properties, functional Infrared imaging (fIR) provides a dynamical and functional indirect evaluation of local haematic flow, thermal properties and the functionality of thermoregulatory effectors of the cutaneous tissue in both basal conditions or in response to stimuli [6, 7]. Many inflammatory joint diseases such as Rheumatoid Arthritis (RA) and Juvenile Arthritis have been studied with fIR [8, 9]. In RA for example a direct relationship between disease activity (Ritchie score, morning stiffness) and skin temperature as for the heat distribution index was demonstrated. Several IR imaging studies have been performed to differentiate PsA plaque skin [1, 4, 10]. However, to our best knowledge, no study with the exception of our pilot study (Capo et al., [11]) has ever been performed to study the thermal changes of skin overlying joint in PsA that may be manifested by the PsA inflammatory condition that may present on the distal interphalangeal joints as well as larger joints. Moreover, while most of the IR diagnostic studies of PsA were usually performed on the basis of static IR evaluation (without performing any Challenge/diagnostic test) of the abnormalities in the corresponding thermal pattern [1, 4, 10], a dynamic and functional IR evaluation of temperature changes of skin overlying the proximal and distal interphalangeal Joints of PsA patients in both basal conditions or in response to functional (isometric) exercise, is rare. Studies have shown that skin blood flow (and thus indirectly cutaneous temperature) during isometric exercise undergoes a limitation due to cutaneous vasoconstriction [7]. Recently, isometric exercise was evident to be potentially able to elicit significantly different thermal responses in both healthy and PsA patient groups [12]. However, such evidence was based on a qualitative study without providing a broad understanding of the complex mechanism underlying thermoregulation malfunctions in this disease [12]. Therefore, a quantitative evaluation of the cutaneous temperature of the skin overlying the proximal and distal Interphalangeal Joints of PsA patients in both basal conditions and in response to functional (isometric) exercise, could provide a functional indicator of the hypothetical PsA-related thermoregulatory malfunctions of skin overlying joints due to their inflammation thus providing a mean to assess indirectly PsA disease activity and help its primary diagnosis. Recently, control theory has been used to model different thermal responses due to pathological, functional, and morphological alterations in the skin thermoregulation system associated with vascular diseases like Raynaud’ phenomenon (RP) [6, 7, 12, 13]. Ismail et al. [12, 13] adopted a prototype second-order control system to model the skin thermal recovery response to a mild cold challenge. They suggested that the direct estimation of its time domain characteristics could provide an effective description of the local thermoregulatory malfunctions in the percense of RP disease and Varicocele. Mariotti et al. [6, 7] proposed a thermoregulatory model based on a homeostatic negative feedback loop characterized by four distinct functional parameters, which describe how thermal control mechanisms are activated and maintained in response to a cold challenge in the percense of RP disease and Varicocele. Due to the model limitation of the direct estimation approach [12, 13], in this study, we propose to implement the model proposed by Mariotti et al. [6] to evaluate how the PsA joint inflammatory characteristics affect the skin thermal recovery capability in response to isometric exercise. We expect that the application of such model may help in the primary diagnosis of PsA.

## Modeling cutaneous thermoregulatory effectors for isometric exercise

**Figure 1:**
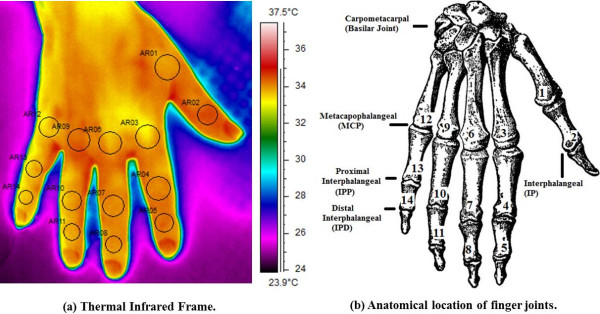
**Selected Areas (AR) for the fourteen Regions of Interest (ROIs) located on the hand’s dorsum corresponding to proximal and distal interphalangeal joints, the metacarpophalangeal joints, nails and inter-bones muscles.** **A)** Thermal infrared frame. **B)** Anatomical location of finger joints.

**Figure 2:**
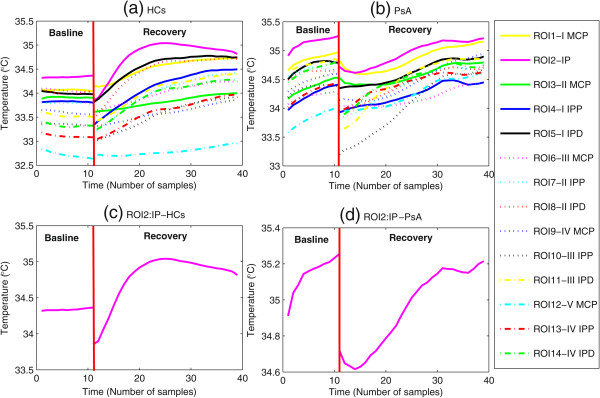
**Temperature vs. time curves obtained from thermal imaging data during baseline, i.e. before performing the isometric exercise and 5 min after the isometric exercise separated by a red vertical line.** (Subjects were required to press every 2 seconds, and for a total of 2 minutes, the dynamometer handle, at the 20% of previously assessed maximal individual strength.) measured from: **(a)** 14 ROIS for HCs subject, **(b)** 14 ROIS for PsA subject, **(c)** the randomly chosen representative ROI2 for HCs subject, and **(d)** the randomly chosen representative ROI2 curve for PsA subject.

Cutaneous circulation is a major effector of human thermoregulation [14]. Cutaneous vessels dilate or constrict in response to either thermal stress, i.e. temperature changes, arose exogenously from variations in environmental temperatures or endogenously from the body itself, as occurs during isometric exercise [14, 15]. The initiation of isometric hand grip exercise has a little effect on the cutaneous circulation in nonglabrous (hairly) skin, whereas the activation of vasodilator system at skin overlying the PSA inflamed joint, causes vasoconstriction due to withdrawal of the active vasodilator activity [16, 17]. In PsA, heat production of active joint, muscle and elevation of inflammatory blood flow in tendons (as shown usually by Eco power Doppler in this disease [1]), may increase the finger’s skin temperatures [15] (as visually evident in figure two). In fact, according to Johnson and colleagues [15] many factors can modulate control mechanisms of the cutaneous vasculature, such as gender, aging, and clinical conditions. Cutaneous vasoconstriction and vasodilation are vasomotor responses mediated by a sympathetic control action from the simulated temperature regulating center in the anterior hypothalamus [14]. Homeostasis is basically maintained by a negative feedback loop, similar to a thermostat [18], which regulates the energy exchange with the environment at the cutaneous level through metabolic and hemodynamic processes that determine finger temperature at any given time [19]. Employing Control System Theory, the homeostatic process can be seen as a feedback controlled system. This kind of system considers a reference signal to produce the desired output. The reference signal indicates the value that the output has to assume. The reference value is represented by superficial basal temperature that can be considered steady during the experiment, while the output is the superficial finger temperature. The controlled isometric exercise induces a finger temperature (plant controlled output) change from the basal value (reference value). The difference between the plant controlled output and the reference value (i.e., the output error) prompts the thermoregulatory reaction in order to restore the basal value by steering the output error to zero. The time-evolution of the finger temperature can be recorded by means of thermal IR imaging [6, 7, 11–13]. Examples of temperature versus time curves, captured at finger joints (shown in Figure 1) are reported in Figure 2. According to Control System Theory, differences in the temperature recovery curves depend on the efficacy of the cutaneous thermoregulatory effectors, which in turn can be represented by the actual values of a given set of functional modeling parameters.

### Problem statement

**Figure 3:**
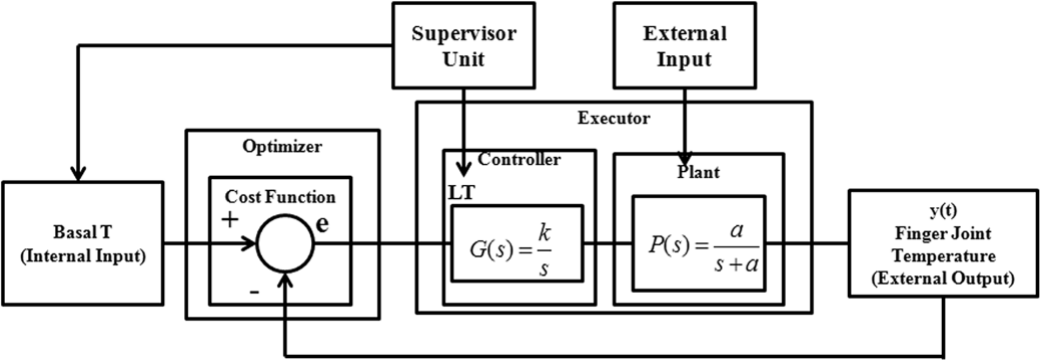
**The overall architecture of the thermoregulatory system (adapted to [** [6] **]).**

Experimental evidence (Figure 2) showed that finger cutaneous thermoregulatory response after the isometric exercise for PsA plaque skin regions has different dynamic characteristics with respect to the healthy skin regions [11]. The wide number of complex processes potentially involved in temperature control and in its alteration suggests considering the overall control system as a ‘black box’, whose overall structure can be investigated by analyzing the input-output time responses either in the healthy or in the pathological conditions [6, 7, 12, 13, 20]. Mariotti et al. [6] proposed a feedback thermoregulatory model through two hierarchical control units: a higher level unit (supervisor) and a feedback lower level executor, driven by the supervisor as shown in Figure 3. These two hierarchical control units were proposed to model both local/peripheral, and systematic/central thermoregulatory effectors known to respond to the isometric exercise attempting to restore the basal temperature [16]. In fact, the supervisor sets the reference signal on the basis of the basal pre-stress temperature and the onset time. The overall performance of the thermoregulatory effecting processes depends on the activity of both the supervisor and the executor. Besides the contribution of the thermoregulatory effector mechanisms, the finger temperature (i.e., system output) is also influenced by the thermal exchange between the finger and the surrounding environment. This thermal exchange depends on the temperature difference which constitutes the external input to the thermoregulatory system [18, 19].

### Model structure

Figure 3 shows the overall architecture of the model proposed by Mariotti et al. [6]. The only observable output is the finger cutaneous temperature [6, 7] y(t), obtained through thermal IR imaging. No information about internal variables is available. Some assumptions can be made about the general structure and the order of the thermoregulatory control system identified with a grey box approach, with the aim of introducing functional parameters to both quantitatively and qualitatively describe the thermoregulatory effector mechanisms [21]. The system is characterized by an external input (room temperature) and a steady state regime reference signal (r) (basal finger cutaneous temperature T). The reference signal can be measured by IR imaging before the initiation of the isometric exercise, and averaged over time to provide a constant reference value T. Visual inspection of the thermal recovery after the isometric exercise confirmed that skin thermoregulatory cutaneous effector system could be assumed as a second-order time-invariant feedback system [22]. In particular, the executor (feedback lower level unit) is composed of a controller and a plant block in sequence (Figure 3), both assumed to be time invariant systems described by first-order transfer functions. Therefore, the plant output y(t) (i.e., the finger temperature) is governed in the time domain by the following differential equation:
1

Where u is the plant input, a and b are constant coefficients. The post-exercise temperature y(0) (i.e., the temperature measured immediately after the end of the isometric exercise) constitutes the initial condition for the response of the control system. The plant input u (t) is then the sum of the feedback controller output m (t) plus the additional external input d as shown in Figure 3:
2

Input d represents passive heat exchange with the environment. Therefore, it depends on room temperature and y(t). In other words, input d can be seen as the uncontrolled effect of environmental conditions on the finger temperature [6]. The feedback controller block generates the signal m(t) stimulated by the difference between the system output and the reference signal r, namely output error e(t):
3

The feedback controller acts on the plant by the signal m(t) to steer the output error to zero. Common approaches for modeling homeostatic processes are based on an integral-type feedback controller system, which nullifies step-wise variation of the error signal [23]. The differential equation that describes the controller behavior in the time domain is:
4

Where K is a proportionality constant. The supervisor unit activates this controller by means of logic signals (on/off transition). When the supervisor unit logical output is “on”, the feedback is closed on the integral type controller and then the active temperature recovery can start. Otherwise, when the supervisor unit logical output is “off” (during the lag time LT), the controller is disabled to restore the initial condition, while the external input d is independent of this switching logic. The evolution of the system can be described more easily in the Laplace domain. The Laplace transform (L-transform) was performed with the assumptions : i) zero initial conditions y(0), and ii) the plant is unitary gain process with b =a in eq.1 [24], since allowing for gain both the plant and the inputs to the controller would result in degenerate parameters [6].Therefore, the overall model works in open loop for the time instance t <LT [6]:
5

and in closed loop for the time instance t >LT [6]:
6

Where s is the Laplace variable, Y(s), r and d are the output, reference input, and the disturbance inputs, respectively. Moreover, the set of parameters (i.e. a, k, d, and LT) could provide an insight on the dynamics and activity level of thermoregulatory effector mechanisms during both healthy state and the presence of a disease. In fact, the reciprocal of the plant time constant (a) represents the speed of the response of the thermal process to external and internal stimuli. The integral gain (k) could be considered as a descriptor of an active and systemic vasodilation process in restoring and maintaining the reference basal temperature conditions [6], since it refers to the control action and determines the efficiency of the feedback control system in achieving the steady state. The disturbance input (d) represents a passive heat exchange with the environment and, therefore, depends on room temperature and y(t). LT is a time required for the thermoregulatory processes to access the internal re-warming process after the end of the isometric exercise. During this time, the thermal variations are mostly attributable to the passive heat exchange with the environment. Once LT is finished, there is the onset of the re-warming process and the controller starts to restore the reference basal conditions T.

Since the purpose of applying control theory is to offer a model that can fit the sample data well, which means making the calculated system output y* approach the actual/experimental system output y^e^ as closely as possible. The closer those two values are, the better the fitting effect will be. Therefore, the least squares criterion function f [25] can be taken as the fitness function:
7

Where  is the vector of experimental finger re-warming curves’ data points and  is the vector of the estimated model’s data points. The data points are defined from i =1 to number of data points NE, and is the vector of the model parameters, i.e. a, k, d, and LT. From equations 5 and 6, the finger thermoregulatory model (Figure 3) is uniquely described by a, k, d, and LT, which can be estimated based on measurements of T and y(t) [6, 7] by solving the optimization problem defined by the cost function stated in equation 7.

## Materials and methods

### Subjects

11 PsA patients and 9 healthy controls, matched for sex and age, participated in the study. All subjects signed the informed consent form prior to be enrolled for the study, which was approved by the local ethical board. The control subjects did not report any personal or family history of Psoriasis or PsA. Demographic data of the participating subjects are summarized in Table 1. The diagnosis of PsA was performed according to CASPAR criteria [1]. PsA patients had been treated in the past with standard therapies (MTX or CsA) with poor results in terms of pain resolution and quality of life improvement and were eligible for biologic therapy. All the subjects observed a washout period of two weeks before the measurements and were free of any medication that could interfere with the fIR imaging measurements.

**Table 1:** **Demographic data**

Item	HCs	PsA
No. of subjects	9	11
Age (Mean ±Std) (Years)	51 ±13.5	52 ±15.5
Gender (Female/Male)	5/4	5/6

This study was approved by the Human Board Review and conducted according to the Helsinki’s Declaration. All the subjects signed an informed consent and could withdraw from the study at any moment.

### Data collection

A total of 280 experimental temperature curves from 14 regions of interest were collected. Each curve included a baseline and a recovery time-course after a controlled isometric exercise. 154 and 126 curves were collected from PsA patients and HCs, respectively. Selected Areas (AR) for the fourteen Regions of Interest (ROIs) located on the hand’s dorsum corresponding to the interphalangeal joints (IP): both proximal and distal (IPP and IPD respectivily), metacarpophalangeal joints (MCP), nails and inter-bones muscles, as shown in Figure 1. Thermal IR imaging measurements were performed in a controlled-climate room. Patients seated with both hands placed on a table covered with a black sheet; measurements were made on the dominant hand to minimize potential bias due to muscle hypertrophy and motor capabilities. Prior to starting the thermal IR imaging recordings, the patients observed a 20-minute acclimatization period in the recording room, which was set at a standardized temperature (23°C), humidity (50-60%), without any direct ventilation [8]. The subjects were asked to abstain from assuming any vasomotor substance (e.g., alcohol, coffee, tea etc), nor undergo of physical activity during the 2 hours prior to evaluation. High-resolution digital thermal images of the hand were acquired at baseline and after a functional exercise. The exercise consisted of repeated isometric contractions through the compression of a calibrated digital dynamometer interfaced to an ADInstruments 8/30 PowerLab computerized system [7]. Subjects first underwent 1.5 minutes of baseline thermal recording. Next, subjects were required to press the dynamometer handle every 2 seconds for a total of 2 minutes at 20% of their previously assessed maximal individual strength. Soon after the exercise, the subjects repositioned their hand in the starting position, undergoing 5 minutes of thermal recording. We used a 14-bit digital thermal camera (FLIR SC660 QWIP, Sweden), sensitive in the 7-14 *μ* m band and with 0.04 *κ* temperature resolution. The thermal imaging’s sampling rate frequency was set to 0.1 Hz. ROI temperature data were extracted by means of the FLIR ThermaCAM Researcher Professional 2.9. Software.

### Data analysis

### Data preprocessing

**Figure 4:**
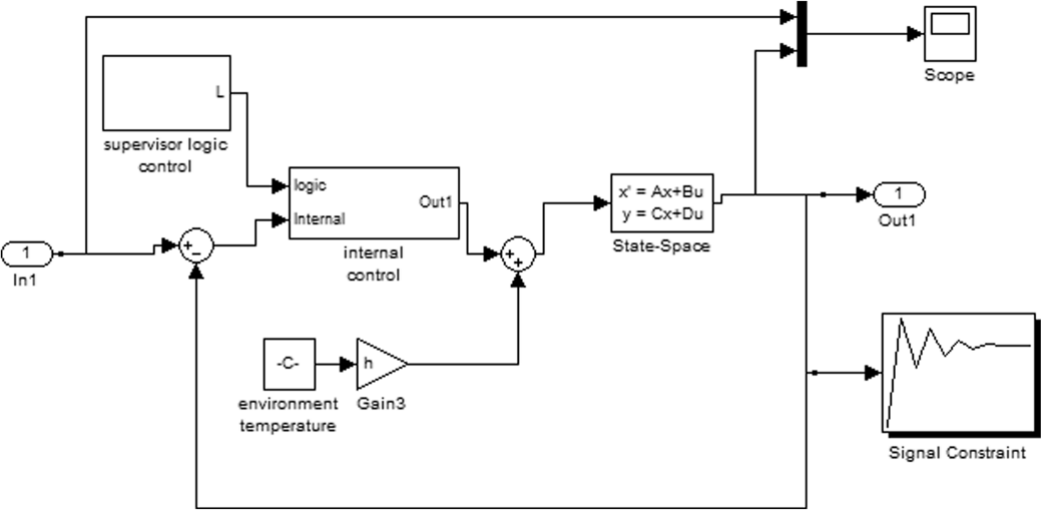
**Thermoregulatory model Simulink block diagram (adapted to [** [6] **]).**

In-home software implemented within the MATLAB^Ⓒ^ platform (http://www.mathworks.com) was used for data and graphic analysis. The time-course of the temperature data was filtered through a smoothing algorithm (span = 5 samples). The control model was implemented the Matlab Simulink Graphical User Interface^Ⓒ^ (Figure 4 shows the implemented Simulink model). Thermoregulatory model responses were simulated by the variable step ODE45 (Dormand-Price) solver.

### Parameter estimation procedure

In order to compute and optimize the four unknown model parameters (a, k, d, and LT), several parameter estimation procedures were performed based on the following optimization theory: i) The initial estimation of the model parameters was assigned based on the graphical approach for real time process-identification [26]. Table 2 summarizes the definition of model parameters using the graphical method; ii) The search space for the model parameters (see Table 3) were selected based on both common limits used in literature [27] and operator evaluation by performing unconstrained simplex optimization method [28] (see Figure 4), considering the system output signal as its reference signal; iii) Since the function to be minimized in the parameter estimation procedure is nonlinear (see equation 3), the corresponding nonlinear least square algorithm used could actually produce local results; therefore the model parameters (a, k, d, and LT) were computed and optimized through the Matlab Simulink Parameter Estimation Toolbox^Ⓒ^, by using a Non-Linear Least Square algorithm [29], while T and y(t) were directly estimated on the thermal IR imaging data.

**Table 2:** **The Time- domain parameter Identification based on graphical method [** [26] **]**

Symbol	Parameter name	Calculation description
K	Process gain*	Process gain is determined by dividing the steady state output (t → *∞*)
		(assumed to be the final output value of y(t)) by the input set point value (T).
LT	Lag time	The lag time or dead time is the time interval between the input being applied to the system
		and the output responding to this signal. The time delay from the onset
		of the re-warming process and the end of the isometric exercise is often
		referred to as lag time (LT) [6].
a	Open loop pole location	It is the inverse of system time constant.
		The system time constant is the time taken for the output
		to reach 63% of the final value.

*Integral Control gain was used to study the whole model gain, as we adapted the process transfer function to be unitary gain one [6].

**Table 3:** **Parameter search space**

Parameter	Minimum	Maximum
Lag Time LT	0	22
Open pole location a	0.01	30
Integral controller gain K	-5	100
Disturbance gain d	-5	10

### Statistical analysis

For each subject, the model parameters were computed for each of the fourteen regions of interest. The statistical analysis was performed to search for the most significant joint regions that could differentiate between PsA patients and HCs based on the estimated model parameters. To this goal, we analyzed the estimated model parameters at each joint regions individually and at the fourteen ROIs all together.The distributions of the estimated model parameters for each group were tested for normality by visual inspection of the frequency distribution and Shapiro-Wilk test [30]. All the parameters for each group were compared through Wilcoxon-Mann-Whitney test [31]. The level of statistical significance was fixed at 0.05. A multiple logistic regression classification algorithm [31] was performed in order to evaluate which parameter better reproduces the probability to detect and classify the presence of PsA as clinically diagnosed, according to the CASPAR criteria [1]. The clinical diagnosis was adopted as independent variable. The classification procedure was a region-based classification. The cut-off for the best classification was established by means of a receiver operating characteristic (ROC) analysis [32] applied to the multiple logistic regression model output. ROC analysis allows the evaluation of the optimal cut-off for a binary classification resulting from a compromise between the 1-specificity, i.e., the false-positive rate, and the sensitivity, i.e., the true positive rate [32].

## Results

**Figure 5:**
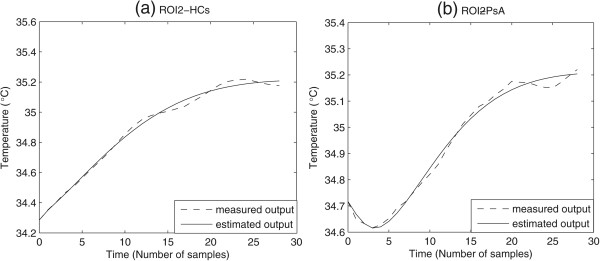
**Comparison between experimental data and identified model response for the studied groups.** **(a)** Healthy control HCs. **(b)** Psoriasis patient PsA.

Figure 5 shows a comparison between the identified response and the experimental temperature curves for two representative cases randomly chosen from PsA and HCs groups, respectively. For all subjects, the IR curves estimated with a minimum cost function higher than 1 were excluded from the statistical analysis in order to guarantee high accuracy of the result. This exclusion occurred in 20 (13%) and 5 (0.04%) curves from PsA and HCs set, respectively.

The distributions of the average model parameters allowed to reject the null hypothesis of the normality test with significant level <0.05. Group mean and standard deviation (Std) for each estimated parameter for each joint region for each group are reported in Table 4.

**Table 4:** **Group average values**

Group	Parameter (Mean(Standard deviation))			
	LT	a	d	K
ROI 1 : I MCP				
PsA	4(6)	0.9(3)	0.04(0.1)	0.9(1.7)
HCs	6(7)	0.1(0.2)	-0.07(0.09)	0.3(0.6)
ROI 2 : IP				
PsA	0.5(0.8)	0.1(0.1)	-0.02(0.1)	0.3(0.4)
HCs	2(3)	0.1(0.17)	-0.02(0.2)	-0.5(1.6)
ROI3 : II MCP				
PsA	3(4)	1(3)	-0.01(0.2)	0.48(1)
HCs	4(4)	0.08(0.08)	-0.03(0.1)	0.4(0.8)
ROI4 : I IPP				
PsA	5(6)	0.9(2)	-0.06(0.1)	0.1(0.1)
HCs	3(3)	2(3)	-0.06(0.08)	0.02(0.04)
ROI5 : I IPD				
PsA	4(5)	0.5(1)	0.03(0.19)	0.7(1.5)
HCs	8(7)	0.4(0.6)	0.007(0.2)	0.4(0.9)
ROI6: III MCP				
PsA	3(5)	0.12(0.17)	-0.01(0.17)	0.3(0.9)
HCs	2(2)	1(1.5)	-0.04(0.04)	0.03(0.07)
ROI7: II IPP				
PsA	3(3)	0.8(2)	-0.1(0.3)	0.2(0.16)
HCs	7(9)	0.2(0.2)	-0.04(0.07)	0.12(0.16)
ROI8: II IPD				
PsA	5(7)	1(3)	-0.1(0.4)	(0.3(0.4)
HCs	4(4)	0.2(0.3)	-0.01(0.2)	0.2(0.3)
ROI9: IV MCP				
PsA	4(4)	0.1(0.09)	-0.007(0.06)	0.14(0.15)
HCs	4(5)	0.6(1.7)	-0.06(0.15)	-0.2(0.4)
ROI10 : III IPP				
PsA	3(4)	0.6(0.9)	-0.06(0.2)	0.5(1.3)
HCs	4(7)	0.2(0.3)	-0.2(0.2)	0.4(1.9)
ROI11 : III IPD				
PsA	3(6)	2(2)	-0.05(0.1)	0.07(0.05)
HCs	3(3)	0.1(0.18)	-0.08(0.1)	0.15(0.1)
ROI12 : V MCP				
PsA	7(8)	0.8(1)	0.03(0.1)	1.5(3)
HCs	3(4)	0.6(1.5)	(0.01(0.3)	0.9(1.7)
ROI13 : IV IPP				
PsA	3(7)	1(2)	0.04(0.2)	0.1(0.1)
HCs	4(4)	0.9(2)	0.02(0.1)	0.02(0.05)
ROI14 : IV IPD				
PsA	6(7)	0.3(0.4)	-0.01(0.3)	0.1(0.2)
HCs	1(2)	0.15(0.17)	-0.02(0.5)	-0.1(0.9)
All 14 ROIs				
PsA	0.09(0.1)	0.7(2)	-0.02(0.2)	0.4(1)
HCs	4(5)	0.5(1)	-0.04(0.2)	0.2(1)
MCP used for Metacapphalangeal, IP used for Interphalangeal,				
IPP used for proximal interphalangeal,and IPD used for distal interphalangeal				

Wilcoxon-Mann-Whitney result (Table 5) showed statistical significant difference for at least one model parameter between PsA and HCs when studying the 14 joint regions all together and individually when studying Interphalangeal region ROI 2, Metacapophalangeal ROIs 6 and 9. Other joint regions did not show any statistical significance between the groups; therefore we excluded it from the multinomial logistic regression classification.

**Table 5:** **Wilcoxon statistical result**

Region of interest	Model parameter (Ranksum,Z statistics, p value)			
(ROI)	LT	a	d	k
ROI2	(67,-, >0.05)	(88,-, >0.05)	(96,-, >0.05)	(118,-, <0.05)*
ROI6	(100,-, >0.05)	(116,-, <0.05)*	(89,-, >0.05)	(76,-, >0.05)
ROI9	(89,-, >0.05)	(88,-, >0.05)	(68,-, >0.05)	(58,-,<0.05)*
All 14 ROIs	(16050,1, >0.05)	(15142,0.04, >0.05)	(13493,-2.7, <0.05)*	(13047,-3.5, <0.05)*

*means statistically significant.

- means not available; Wilcoxon Mann Whitney test could not measure the z-statistics for small sample size.

**Figure 6:**
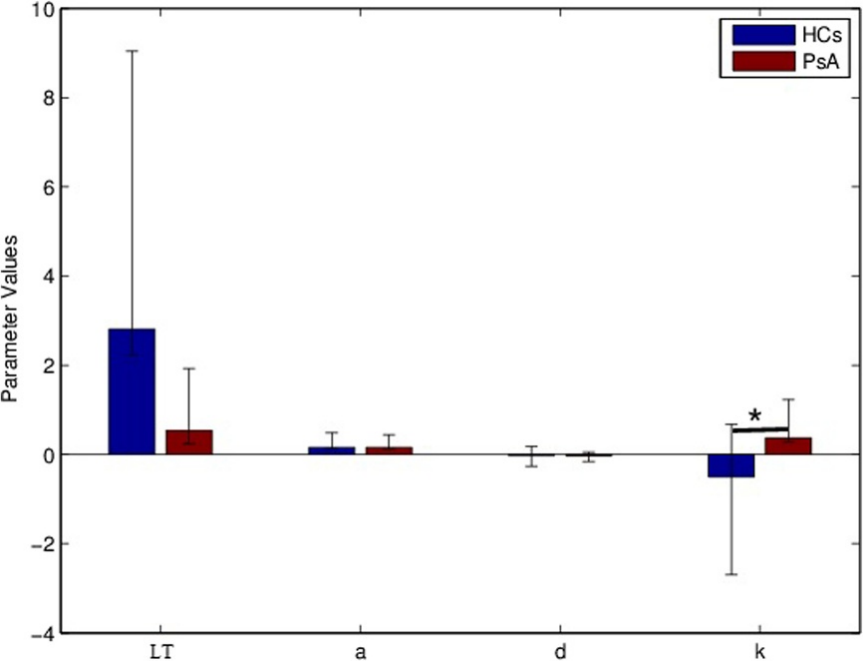
**Bar graph with means and 95% confidence intervals error bars.** Comparison of the group average parameters among groups at the thumb interphalangeal joint region. The horizontal bar with asterick indicates the statistical significance (p <0.05).

Statistically significant differences between PsA and HCs were found in the open loop location a,the disturbance gain parameter d and the integral controller gain k (see Table 5). The PsA group showed the highest values for d and k (see Table 4). The model of multiple logistic regression for the region classification includes one equation for PsA with respect to HCs, with all the estimated parameters LT, a, d and k. The thumb Interphalangeal joint region is the only joint region set that provides a multilogistic regression model that better reproduces the probability to detect and classify the presence of PsA as clinically evaluated. Table 6 reports the estimation of the predictor coefficient (*β*) with its Standard Error (SE), the Wald statistics and the odds ratio of response variable (Exp(*β*)) with respect to the predictor coefficient for the interphalangeal joint. The Wald statistics validate the positive correlation between the model parameter k and the presence of PsA disease. Means with 95% confidence intervals error bars for each parameter for each group are shown in Figure 6.Means with 95% confidence intervals error bars for each parameter for each group are shown in Figure 6.

**Table 6:** **Discriminant parameters for thumb interphalangeal joint region classification**

Model equation	Parameter	***β***	Standard error SE	Wald	DF	sig	Exp( ***β*** )
PsA-HCs	Intercept	-1.7	1	-1.6	1	0.1	0.17
	L	-0.7	0.6	-1.2	1	0.2	0.46
	a	5.5	5	1	1	0.2	24.9
	d	2.3	3.5	0.6	1	0.5	10.6
	k	12	4.7	2.5	1	0.01*	100000

*means statistically significant.

**Figure 7:**
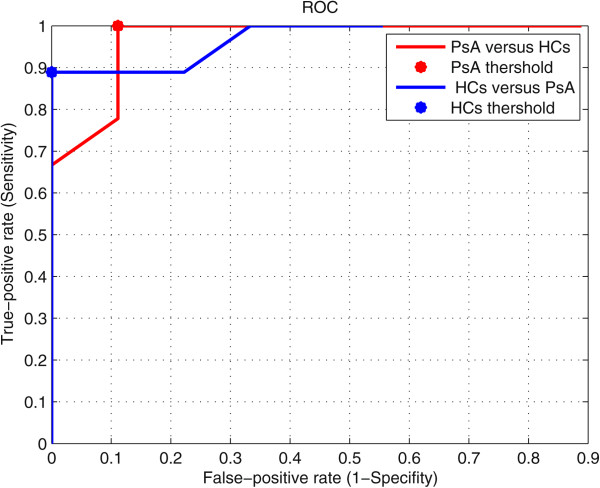
**Roc analysis result using thumb interphalageal joint region.**

The ROC analysis (Figure 7) established one optimal cut-off at 0.4 in order to discriminate between PsA and HCs groups. It provided a true-positive prediction for PsA patients from HCs of 100% and 88.89% for discriminating healthy controls from PsA. Table 7 illustrates the confusion matrix for the region classification.

**Table 7:** **Confusion matrix**

Original group	Predicted group		Correctly classification%
	PsA	HCs	
PsA	100	0	100
HCs	11.11	88.89	88.89

## Discussion

The aim of the present study was to identify quantitative parameters, which describe the functional differences in the thermal recovery of the skin, overlying the proximal and distal interphalangeal joints, from a controlled mild isometric exercise shown by healthy controls and PsA patients [15]. We hypothesized that the implementation of the functional thermoregulatory model proposed by Mariotti et al. [6] to model the skin thermoregulatory processes in response to isometric exercise could evaluate how the pathophysiological differences due to joint inflammatory characteristics corresponding to the PsA disorder affect these processes [9, 15, 17]. The thermoregulatory system was modeled through two hierarchical control units: a higher level unit (supervisor) and a lower feedback level (executor) driven by the supervisor. The implemented model is unequivocally identified by a set of four functional parameters (a, k, d, LT) [6]. The statistical analysis was performed for all the Interphalangeal, Metacarpophalangeal, Proximal Interphalangeal and Distal Interphalangeal regions individually and for the fourteen regions all together, in order to check which of them is the most significantly discriminating region between PsA and HCs groups. Our analysis confirmed that PsA patients exhibit different thermoregulatory dynamic responses to the controlled isometric exercise compared to HCs. Delayed and prolonged re-warming processes characterized by an undershoot onset after the end of the isometric exercise was found [6]. This finding is expressed by the PsAs’ higher values for model disturbance gain (d). Therefore, in the presence of the disease, the skin thermoregulatory recovery process could be mainly based on the passive heat exchange because of the withdrawal of the cutaneous vasodilation activity and the intact vasoconstrictor action in the affected joint region [15, 17]. The higher k values found for PsA with respect to HCs values reflect the higher active and systemic vasodilation after the end of the onset undershoot. This finding might be attributed to a higher emissivity of the PsA areas [4] in the presence of more arterioles or even chronic structure widening of the existing arterioles [5]with higher basal flux [3]. Moreover, PsA showed a faster temperature increase after the undershoot onset compared to the healthy. This finding is evident by the higher mean values of the model parameter of open loop location (a). Region classification on the basis of the model parameters seems to indicate that thumb’s interphalageal joint region is the most expressive region. However, the small sample size does not allow to draw any conclusion about, as further studies on larger samples are needed. The misclassified healthy regions were attributed mostly to those exhibiting very small undershoot recovery curves in response to the controlled exercise. That finding might be due to the little effect on blood flow in nonglobrous skin (finger skin) known to be in normothermic conditions after the end of isometric exercise [15, 16]. It should be pointed out that the implementation of such an approach is valid within two limits: i) the limitations of the model itself, which is the assumption of a step response and the adoption of a simple prototype second-order system, ii) the limit of the time period after the end of the isometric exercise selected to study the dynamics of the temperature recovery curves (i.e. in our case 5 min). The method specificity has to be tested by increasing the number of participants.

## Conclusion

In this study, we identified four quantitative parameters to describe the functional differences in thermal recovery from a controlled isometric exercise shown by PsA and healthy subjects. A homeostatic negative feedback loop, characterized by the four parameters, describes how the control mechanisms are activated, maintained in healthy individuals and impaired in PsA patients. Region classification on the basis of the model parameters demonstrated that Thumb’s interphalageal joint region is the most indicative region for PsA joint inflammatory disease, while further studies on larger samples are needed. In fact, it provided 100% true-positive discrimination for PsA affected regions and 88.89 % of correct classification of healthy regions.

## References

[1] Boehncke WH, Merolal JF, Thaçi D, Krueger GG, Walsh J, Kim N, Gottlieb AB (2014). **Diagnosing and treating psoriatic arthritis-an update**. Br J Dermatol.

[2] Abd El Baky AM, Waked IS (2011). **Non-steroidal anti-inflammatory phonophoresis versus topical application in improvement of hand grip strength in psoriatic arthritic patients**. J Am Sci.

[3] Hern S, Stanton AW, Mellor R, Levick JR, Mortimer PS (1999). **Control of cutaneous blood vessels in psoriatic plaques**. J Investigat Dermatol.

[4] Warshaw TG (1973). **Thermal studies in psoriasis**. J Investigat Dermatol.

[5] Zalewska A, Gralewicz G, Owczarek G, Wiecek B (2005). **Thermography in psoriasis vulgaris evaluation**. Conf Proc IEEE Eng Med Biol Soc.

[6] Mariotti A, Grossi G, Amerio P, Orlando G, Mattei PA, Tulli A, Romani GL, Merla A (2009). **Finger thermoregulatory model assessing functional impairment in raynaud’s phenomenon**. Ann Biomed Eng.

[7] Mariotti A, Di Carlo L, Orlando G, Corradini ML, Di Donato L, Pompa P, Iezzi R, Cotroneo AR, Romani GL, Merla A (2011). **Scrotal thermoregulatory model and assessment of the impairment of scrotal temperature control in varicocele**. Ann Biomed Eng.

[8] Spalding SJ, Kwoh CK, Boudreau R, Enama J, Lunich J, Huber D, Denes L, Hirsch R (2008). **Three-dimensional and thermal surface imaging produces reliable measures of joint shape and temperature: a potential tool for quantifying arthritis**. Arthritis Res Ther.

[9] Devereaux MD, Parr GR, Thomas DP, Hazleman BL (1985). **Disease activity indexes in rheumatoid arthritis; a prospective, comparative study with thermography**. Ann Rheum Dis.

[10] Castillo-Martínez C, Valdes-Rodríguez R, Kolosovas-Machuca ES, Moncada B, González FJ (2013). **Use of digital infrared imaging in the assessment of childhood psoriasis**. Skin Res Technol.

[11] Capo A, Merla A, Mattei P, Auriemma M, Panarese F, Celletti E, Abate M, Romani GL, Amerio P (2013). **Assessment of psoriatic arthritis by means of functional infrared imaging: A pilot study**. Clinc Drug Investig.

[12] Ismail E, Orlando G, Corradini ML, Amerio P, Romani GL, Merla A (2014). **Differential diagnosis of raynaud’s phenomenon based on modeling of finger thermoregulation**. Physiol Meas.

[13] Ismail E, Orlando G, Pompa P, Gabrielli D, Di Donato L, Cardone D, Merla A (2014). **Time-domain analysis of scrotal thermoregulatory impairment in varicocele**. Front Physiol.

[14] Sawasaki N, Iwase S, Mano T (2001). **Effect of skin sympathetic response to local or systemic cold exposure on thermoregulatory functions in humans**. Auton Neurosci.

[15] Johnson JM, Minson CT, Kellogg DL (2014). **Cutaneous vasodilator and vasoconstrictor mechanisms in temperature regulation**. Compr Physiol.

[16] Shibasaki M, Secher NH, Johnson JM, Crandall CG (2005). **Central command and the cutaneous vascular response to isometric exercise in heated humans**. J Physiol.

[17] Johnson JM (2010). **Exercise in a hot environment: the skin circulation**. scand J Med Sci Sports.

[18] Charkoudian N (2003). **Skin blood flow in adult human thermoregulation: how it works, when it does not, and why**. Mayo Clin Proc.

[19] Sanial DC, Maji NK (2001). **Thermoregulation through cutaneous under variable atmospheric and physiological conditions**. J Theor Biol.

[20] Merla A, Di Donato L, Di Luzio S, Romani GL (2002). **Quantifing the relevance and stage of disease with the tau image technique: a complementary diagnostic imaging technique based on infrared functional imaging**. IEEE Eng Med Biol Mag.

[21] Oussar Y, Dreyfus G (2001). **How to be a gray box: dynamic semi-physical modelling**. Neural Netw.

[22] Rollins D, Bhabdar N, Hulting S (2006). **System identification of the human thermoregulatory system using continuous-time block-oriented predictive modelling**. Chem Eng Sci.

[23] Waterhouse (2004). **Homeostatic control mechanism**. Anaesth Intensive Care.

[24] Forssel U, Ljung L (1999). **Closed loop identification revisited**. Automatica.

[25] Nocedal J, Wright SJ (2006). Numerical Optimization.

[26] Kealy T, O’Dwyer A, Smith Y (2001). **Comparison of open and closed loop process identification techniques in the time domain**. Proceedings of the 3nd Wismarer Automatisierungssymposium, Wismar, Germany, September, 2001, Paper 1.3-4.

[27] Luo Y, Wang Y, Kong L (2008). **System identification of thermal process based on chaos particle swarm optimization**. Automation and Logistics, 2008. ICAL 2008. IEEE International Conference on.

[28] Lagarias JC, Reeds JA, Wright MH, Wright PE (1998). **Convergence properties of the nelder-mead simplex method in low dimensions**. SIAM J Optimizat.

[29] Tarantola A, Valette B (1982). **Generalized nonlinear inverse problems solved using the least squares criterion**. Rev Geophys Space Phys.

[30] Razali NM, Wah YB (2011). **Power comparisons of shaprio-wilk, kolmogorov-smirnov, lilliefors and anderson- darling tests**. J Stat Model Analytics.

[31] Agresti A (2002). Categorical Data Analysis.

[32] Westin LK: **Receiver operating characteristic (roc) analysis:evaluating discriminance effects among decision support systems****.** Technical report, Department of Computing Science in Umeå University, Computer Science Department,Umeå, Sweden; 2001. [http://www8.cs.umu.se/research/reports/2001/018/part1.pdf]

